# Antenatal depression: an examination of prevalence and its associated factors among pregnant women attending Harare polyclinics

**DOI:** 10.1186/s12884-020-02887-y

**Published:** 2020-04-06

**Authors:** M. Kaiyo-Utete, J. M. Dambi, A. Chingono, F. S. M. Mazhandu, T. B. Madziro-Ruwizhu, C. Henderson, T. Magwali, L. Langhaug, Z. M. Chirenje

**Affiliations:** 1 Department of Psychiatry, College of Health Sciences, University of Zimbabwe, P O Box A178, Harare, Zimbabwe; 2 African Mental Health Research Initiative (AMARI), College of Health Sciences, University of Zimbabwe, P O Box A178, Harare, Zimbabwe; 3 Department of Rehabilitation, College of Health Sciences, University of Zimbabwe, P O Box A178, Harare, Zimbabwe; 4 Department of Psychiatry, Midlands State University, P Bag, 9055 Gweru, Zimbabwe; 5 Department of Health Services and Population Research, Institute of Psychiatry, Psychology and Neurosciences, King’s College London, Strand, London, WC2R 2LS United Kingdom; 6 Department of Obstetrics and Gynaecology, College of Health Sciences, University of Zimbabwe, P O Box A178, Harare, Zimbabwe

**Keywords:** Antenatal depression, Prevalence, Associated factors, Zimbabwe

## Abstract

**Background:**

Antenatal depression is the most prevalent common mental health disorder affecting pregnant women. Here, we report the prevalence of and associated factors for antenatal depression among pregnant women attending antenatal care services in Harare, Zimbabwe.

**Methods:**

From January–April 2018, 375 pregnant women, aged 16–46 years, residing mostly in Harare’s high-density suburbs were recruited from two randomly-selected polyclinics. Antenatal depression was measured using the Structured Clinical Interview for DSM-IV. Sociodemographic data including; maternal age, education, marital status, economic status, obstetric history and experiences with violence were also collected. Chi-square tests and multivariate logistic regression analysis were used to determine the association between antenatal depression and participants’ characteristics.

**Results:**

The prevalence of antenatal depression was 23.47% (95% CI: 19.27–28.09). Multivariate logistic regression analysis revealed intimate partner violence (IPV) [OR 2.45 (95% CI: 1.47–4.19)] and experiencing negative life events [OR 2.02 (95% CI: 1.19–3.42)] as risk factors for antenatal depression, with being married/cohabiting [OR 0.45 (95% CI: 0.25–0.80)] being a protective factor.

**Conclusion:**

The prevalence of antenatal depression is high with associated factors being interpersonal. Context-specific interventions are therefore needed to address the complexity of the factors associated with antenatal depression.

## Background

Maternal and child health remains a worldwide health priority. Although the global community has worked strenuously to reduce physical causes of maternal and child mortality and morbidity [1], other contributing factors, such as maternal mental health, have received less attention [2–4]. Unfortunately, adverse maternal mental health increases the risks of obstetric and child outcomes such as low birth weight, prematurity and increased risks of assisted births [5–8], and lifelong maternal mental health issues [9]. For example, common mental health disorders in the perinatal period, particularly depression, are highly prevalent [10].

Global prevalence estimates of antenatal depression vary across regions [8]. The burden of antenatal depression is generally higher in low- and middle-income countries (LMICs) [10, 11]. For instance, the prevalence of maternal depression in women from Sub-Saharan Africa ranges from 20 to 49% [5, 12–23]. These prevalence variations can be explained by several reasons. Firstly, the population studied can explain these differences. Studies investigating HIV-positive, pregnant women reported higher prevalence of antenatal depression [20–22]; HIV infection typically increases the risk of depression up to four times [24, 25]. Secondly, pregnancy stage influences prevalence estimates; depression occurrence is trimester-dependent with the burden being greater in the third trimester [13, 26]. Thirdly, tools used to evaluate depression can also explain maternal depression disparities [27]. For instance, most studies in Southern Africa have either used generic depression screening tools [28] or postnatal depression screening tools [29] in measuring antenatal depression which is a potential methodological pitfall. Further, most screening tools are self-report outcome measures which may lead to higher prevalence estimates [10, 27]. On the contrary, diagnostic tools such as the Structured Clinical Interview for Diagnostic and Statistical Manual 4th edition (SCID-IV) [30] which offer a definitive depression diagnosis, are infrequently used. There is only one study in Southern Africa that used a diagnostic tool [17]. Although the Malawian study used a diagnostic tool, the study outcomes may have limited external validity [17]. Structured interviews were carried out by non-mental health practitioners and selection bias was highly probable since participants were recruited using convenience sampling method [17]. There is, therefore, need for robust methodologies in estimating the burden of antenatal depression in low-resource settings. Accordingly, we set to estimate the prevalence of antenatal depression among pregnant women attending Harare polyclinics using the Structured Clinical Interview for DSM-IV (SCID-IV), a diagnostic tool. We also explored potential factors associated with antenatal depression. Understanding the burden of antenatal depression will aid in identifying pregnant women at risk and intervene appropriately thereby reducing its negative effects.

## Methods

### Study setting

The study was conducted in Harare, Zimbabwe at two randomly selected polyclinics run by the Harare City Council Health Directorate. The city primary health care facilities serve a population of lower socioeconomic status. They charge an equivalent of US$25.00 for maternity services covering antenatal, delivery, and postnatal care [31]. The health facilities are staffed by midwives and registered nurses and refer complicated cases to tertiary hospitals. Antenatal care services are offered from Monday to Thursday, and postnatal care on Fridays. Labour and delivery services are available every day [31]. On average, each polyclinic attends to 20–30 pregnant women a day.

### Study design and sample

We followed a cohort of pregnant women from their second trimester through to 6 weeks post-delivery. Baseline data, which we are reporting in this paper, were collected from January to April 2018. All pregnant women in their second trimester, aged 15–49 years, fluent in English or Shona, and were receiving antenatal care at the study sites were included in the study. Women on treatment for severe mental disorders, such as bipolar and schizophrenia according to doctor’s notes, unable to provide written consent, or were unwilling to be followed up postnatally were similarly excluded. Assuming a 39% prevalence of antenatal depression [19], a sample of 366 women was needed to achieve a 5% precision at 95% confidence intervals.

### Data collection

The Structured Clinical Interview for Diagnostic and Statistical Manual for Mental Health version IV (SCID-IV), [30] was used to measure antenatal depression. This diagnostic tool (SCID-IV) gives a definitive diagnosis of depression [30]. It is administered by trained mental health professionals; proficient in the use of open-ended questions, diagnostic evaluations, and have cultural knowledge in a wide range of mental, neurological and substance use disorders. The SCID-IV has three categories for diagnosis of depression: “no depression”, “minor depression” and “major depression”. For the purpose of this analysis, depression status was further dichotomised to “Depressed” for those that had “minor depression” and “major depression”, and “not depressed” for those who had no depression. The tool has been translated and used previously in Zimbabwe to assess postnatal depression [32]. In this study, trained study psychiatrists administered the SCID-IV.

A separate questionnaire covering multiple domains was used to elicit factors associated with antenatal depression. Variables elicited included sociodemographic questions, for example, participant’s age, educational level, and economic status. Obstetric history included questions about the woman’s parity and gravidity, pregnancy-induced illnesses (e.g. hypertension, diabetes), previous obstetric complications (e.g. premature births, stillbirths, neonatal births); use of family planning methods prior to this pregnancy and whether the pregnancy was planned or not. We also asked about the woman’s HIV status, other chronic illnesses, previous history of mental illnesses and treatment related to these illnesses. Psychosocial issues included questions about the woman’s marital status, exposure to intimate partner violence and social support. These questions were loaded onto an android tablet using Open Data Kit [33]. Questionnaires were interview-administered by trained surveyors using a computer-assisted survey instrument (CASI) [34]. All questions were checked for appropriate linguistic comprehension through cognitive interviews prior the main study [35].

On each day of data collection, the principal investigator (MKU) introduced the study to pregnant women waiting for their antenatal care appointments. Prospective participants who met the inclusion criteria were asked to randomly pick an envelope that contained a red or green card from a bucket. Those who picked a green card were selected to participate. Thereafter, participants were invited into a private space where more detailed information about the study was provided. Following written informed consent, the CASI demographic questionnaire was administered. Participants were permitted to continue with the usual processes of her antenatal care appointment whilst awaiting assessment by the study psychiatrist (TMR or FSMM). If diagnosed with major depression, participants were referred to the clinic’s mental health nurse or the Friendship Bench, a brief psychological intervention for common mental disorders delivered by lay health workers [36].

### Data analysis

Data were analysed in STATA 14 software (StataCorp LP, College Station, TX, USA). Descriptive statistics (frequencies and percentages) were used to estimate the prevalence of antenatal depression and to describe the sample’s characteristics. Bivariate analyses were done to identify participant characteristics which were statistically associated with antenatal depression. Thereafter, a multivariate logistic regression model was fitted to identify independent covariates. The strength of association was measured by odds ratios with their 95% confidence intervals, all tests were two-tailed and significance level was set at *p* < 0.05.

### Ethical consideration

Joint Research Ethics Committee for the University of Zimbabwe, College of Health Sciences and Parirenyatwa Group of Hospitals (JREC 158/17) and the Medical Research Council of Zimbabwe (MRCZ /A/2209) approved the study. Permission was granted by the Harare City Health Directorate and the clinic management to carry out the study at the study sites. To maintain confidentiality, Participant Identity Numbers (PIDs) were used in lieu of participants’ names. All interviews were carried out in a private space. Pregnant women aged 15–17 years are considered emancipated minors [37], therefore, were able to give consent to participate in this study. As a token of appreciation for their time, participants were each given a small gift containing toothpaste, soap and petroleum jelly.

## Results

### Participants’ Sociodemographic characteristics and other associated factors

Table 1 shows the participants’ sociodemographic characteristics and other associated factors. Three hundred and seventy-five pregnant women completed both the electronic questionnaire and the SCID-IV. Most participants were aged between 20 and 29 years (*n* = 223; 59.5%), had at least a secondary school level education (*n* = 293; 75.0%), and were unemployed (*n* = 238; 67. 0%). Of the 232 (61.9%) multigravida women, 77 (20.5%) had a previous obstetric complication. Thirty-two (8.5%) had a chronic illness diagnosed in the current pregnancy. All women had been tested for HIV with a quarter (*n* = 93) being HIV-positive. Only 2 (0.5%) had a history of mental illness and 68 (18.13%) had a history of physical chronic illness. More than three-quarters of the participants (*n* = 297) were married/cohabitating with their baby’s father. One third (*n* = 135) had experienced intimate partner violence. Forty-three percent (*n* = 163) had experienced a negative life event. Almost two-thirds (*n* = 241) felt they had some support when they felt overwhelmed with life. After dichotomising the results, 88 (23.5%) women were diagnosed as depressed.

**Table 1:** Sociodemographic characteristics and other risk factors (*N* = 375)

Characteristic	Frequency (%)
Age groups	
< 20 years	56 (14.93%)
20–24 years	118 (31.47
25-29 years	105 (28.00)
30–34 years	49 (13.07)
> =35 years	47 (12.53)
Educational levels	
At least primary school level	20 (5.33)
At least secondary school level	293 (78.13)
Advanced level and tertiary level	62 (16.53)
Residence	
High density	307 (81.87
Low density	50 (13.33
Other	18 (4.80
Occupation	
Not employed	235 (62.67
Self-employed (erratic income)	99 (26.40
Employed (steady income)	41 (10.93)
Gravidity	
Primigravida (first pregnancy)	143 (38.13%)
Multigravida (pregnant before)	232 (61.87)
Planned pregnancy	
Yes	279 (74.40%)
No	96 (25.60%)
History of obstetrics complications	
Never given birth	143 (38.13%)
No previous obstetric complications	155 (41.33%)
Previous obstetric complications	77 (20.53%)
HIV status	
Positive	93 (24.80%)
Negative	282 (75.30%)
Marital status	
Married/cohabitating	297 (79.20)
Other	78 (20.80%)
Experienced a negative life event	
Yes	163 (43.47%)
No	212 (56.53%)
Had someone to talk with when feeling overwhelmed	
Yes	241 (64.27%)
No	134 (35.73%)
Experienced intimate partner violence	
Yes	121 (32.27%)
No	254 (67.73%)

### Associated factors of antenatal depression

Antenatal depression was associated with: having a chronic illness diagnosed during the current pregnancy (*p* = 0.017), being married or cohabitating (*p* < 0.001), not having someone to talk to when feeling overwhelmed with life (*p* = 0.008), having experienced a negative life event in the past year (*p* = 0.001), and having experienced intimate partner violence (*p* < 0.001) (see Table 2). Intimate partner violence [OR 2.45 (95% CI 1.47–4.19)], negative life events [OR 2.02 (95% CI 1.19–3.42)] and being married or cohabitating with baby’s father [OR 0.45 (95% CI 0.25–0.80)] remained statistically significant after multivariate logistic regression. Women who experienced intimate partner violence were 2.5 times more likely to have antenatal depression than those who did not. Those women who had had a negative life event in the past year were twice more likely to have antenatal depression than those who reported no such events. However, being married to or cohabitating with the baby’s father reduced the odds of developing antenatal depression [OR 0.45 (95% CI 0.25–0.80)]. (See Table 3).

**Table 2:** Bivariate analysis showing factors associated with Antenatal Depression, *N* = 375

Characteristics	Overall (*N* = 375)	Depressed (*N* = 88)	Not depressed (*N* = 287)	*p*-value
Age group				0.673
< 20 years	56 (14.9%)	10 (11.4%)	46 (16.0%)	
20–24 years	118 (31.5%)	32 (36.4%)	86 (30.0%)	
24–29 years	105 (28.0%)	22 (25.0)	83 (28.9%)	
30–34	49 (13.1%)	12 (13.6%)	37 (12.9%)	
> =35	47 (12.5%)	12 (13.6%)	35 (12.2%)	
Educational level				0.427
At least primary level	81 (21.6%)	6 (6.8%)	14 (4.9%)	
At least secondary level	260 (69.3%)	71 (80.7%)	222 (77.4%)	
Advanced + tertiary level	34 (9.1%)	11 (12.5%)	51 (17.8%)	
Gravidity				0.521
Primigravida	143 (38.1%)	57 (64.8%)	175 (61.0%)	
Multigravida	232 (61.9%)	31 (35.2%)	112 (39.0%)	
Planned pregnancy				0.071
Yes	279 (74.4%)	29 (32.9%)	67 (23.3%)	
No	96 (25.6%)	59 (67.1%)	220 (76.7%)	
History of obstetric complications				0.814
Never given birth	143 (38.1%)	31 (35.2%)	112 (39.0%)	
No previous obstetric complications	153 (40.8%)	38 (43.2%)	117 (40.8%)	
Previous birth complications	79 (21.1%)	19 (21.6%)	58 (20.2%)	
Chronic illnesses diagnosed in the current pregnancy				0.017*
Yes	32 (8.5%)	13 (14.8%)	19 (6.6%)	
No	343 (91.5%)	75 (85.2%)	268 (93.4%)	
HIV status				0.960
Positive	93 (24.8%)	22 (25.0%)	71 (24.7%)	
Negative	282 (75.2%)	66 (75.0%)	216 (75.3%)	
Marital status				< 0.001*
Married/cohabitating	297 (79.2%)	58 (65.9%)	239 (83.3%)	
Other	78 (20.8%)	30 (34.1%)	48 (16.7%)	
Negative life event				0.001*
Yes	179 (47.7%)	56 (63.6%)	123 (42.9%)	
No	196 (52.1%)	32 (36.4%)	164(57.1%)	
Someone to talk to				0.008*
Yes	134 (35.7%)	67 (76.1%)	174 (60.6%)	
No	231(61.6%)	21 (23.9%)	113 (39.4%)	
Intimate partner violence				< 0.001*
Yes	121 (32.3%)	44 (17.3%)	77 (63.6%)	
No	254 (67.7%)	44 (36.4%)	210 (82.9%)	

*statistically significant at *p* ≤ 0.05

**Table 3:** Multivariate Logistic Analysis showing factors significant associated with Antenatal Depression, *N* = 375

Characteristic	Depressed (*N* = 88)	Not depressed (*N* = 287)	Adjusted OR	95% CI	*p*-value
Chronic illness diagnosed during current pregnant					0.334
No	76 (86.36%)	231 (80.49%)	1		
Yes	12 (13.64%)	56 (19.51%)	1.50	0.65–3.42	
Marital status					0.007*
Other	30 (34.09%)	48 (16.72%)	1		
Married/cohabitating	58 (65.91%)	239 (83.28%)	0.45	0.25–0.80	
Negative life event					0.009*
No	36 (40.91%)	176 (61.32%)	1		
Yes	52 (59.09%)	111 (38.68%)	2.02	1.19–3.42	
Someone to talk to when overwhelmed					0.320
No	21 (23.86%)	113 (39.37%)	1		
Yes	67 (76.14%)	174 (60.63%)	1.35	0.75–2.42	
Intimate partner violence					0.001*
No	44 (36.36%)	77 (63.64%)	1		
Yes	44 (17.32%)	210 (70.38%)	2.48	1.47–4.19	

*statistically significant at *p* ≤ 0.05

## Discussion

The aim of this study was to examine the prevalence and associated factors of antenatal depression among pregnant women in Harare, Zimbabwe. To our knowledge, this is the first study in Zimbabwe looking at antenatal depression in all pregnant women regardless of HIV status. The prevalence of antenatal depression in our study is high i.e. one in four pregnant women were depressed. Salient predictors to antenatal depression were interpersonal in nature: being married or in an informal, steady relationship were protective factors, and having experienced intimate partner violence or a negative life event were risk factors.

The high prevalence rate in this study is comparable to other LMICs [12, 13, 16, 17]. In South Africa, for example, the prevalence of antenatal depression ranges from 20 to 49% [19, 21, 23, 38]. A recent local/Zimbabwean study among HIV-positive pregnant women [22] also showed a higher prevalence of antenatal depression (39.4%). This is unsurprising as given the high depression comorbidity in people living with HIV [24]. Nevertheless, in the aforementioned study, Nyamukoho et al. [22], used a screening tool, the Edinburgh Postnatal Depression Scale (EPDS), to measure depression, instead of a diagnostic tool as used in our study which may explain discrepancies with the current study. Previous studies suggest that depression screening tools overtly yield higher prevalence rates than diagnostic tools [27]. Prevalence rates obtained when using diagnostic tools are more reflective of the burden of antenatal depression since diagnostic tools are more definitive in their diagnosis [27]. Further, the EPDS has been previously validated among Zimbabwean postnatal women only [32], it is not validated among pregnant women in our setting. This may, therefore, account for differences in antenatal depression as revealed in the current study.

Factors associated with antenatal depression, such as intimate partner violence (IPV), experiencing a negative life event, and marital status, are relational. This is consistent with other regional studies [12–23]. In Zimbabwe, recent studies have also shown that intimate partner violence is a risk factor for depression in the perinatal period [22, 39]. Unlike other studies in LMICs [5, 12, 13, 17, 18, 21, 40], we did not find any significant associations of antenatal depression with HIV, maternal age, educational level, history of obstetric complications, social support and gravidity. This may be due to methodological differences.

The results of our study highlight the complexity of marital relationships. Being married/cohabitating had a positive effect on maternal mental health, yet intimate partner violence, which is highly prevalent in marriages [39, 41], was negatively associated with antenatal depression. This complexity, yet an important finding, needs to be understood better when thinking of interventions for antenatal depression. Future qualitative studies maybe warranted to explore this phenomenon further. More importantly, it would be beneficial to look at interventions that not only target the affected women but the whole family. Although there has been repeated calls for male involvement in maternal and child health care [42], uptake remains very low mainly due to the traditional beliefs that maternity issues are females’ expertise [43–45]. Involving male partners in prevention of antenatal depression may potentially play a crucial role since they are at the core of the factors associated with depression as shown in our study.

Given the high rates of antenatal depression and evidence that it has consequences in the post-natal period for both the mother and infant, it is important that culturally sensitive interventions to address these risk factors are available. In Zimbabwe, one intervention, The Friendship Bench, which uses problem solving therapy, has been shown to alleviate depression in the general population [36]. This intervention is currently being rolled out by the Ministry of Health and Child Care in primary care settings. The current scale-up of the Friendship Bench offers a unique opportunity to adapt it so that the intervention tackles the associated factors of antenatal depression seen here.

Our study outcomes need to be interpreted with caution due to some methodological limitations. Social desirability bias is highly likely since the interviews were face-to-face. This may translate to an underestimation of the prevalence of antenatal depression. Additionally, this study was only carried out in urban areas; as such, its generalizability to rural settings may not be guaranteed. Prevalence of antenatal depression varies across trimesters. It would be important to examine depression across trimesters; this study only reports depression across the gestational period. This study also used a diagnostic tool that can only be administered by trained mental health professionals, this may not be practical in our settings where we lack the trained manpower. Despite these limitations, the study adds to the already existing body of knowledge on the burden of antenatal depression. It also helps to inform policy makers on the importance of screening for depression in pregnancy to prevent its negative effects not only on the mother but on the baby as well.

## Conclusion

The prevalence of antenatal depression among pregnant women attending antenatal clinics in Harare is high: one in four pregnant is depressed. Being married/cohabitating was a protective factor against antenatal depression, and having experienced intimate partner violence or a negative life event were risk factors of antenatal depression.

Most women are likely to seek health care services during pregnancy hence the antenatal period offers a great opportunity to diagnose and treat antenatal depression. It is important to screen for depression during pregnancy and manage it since it is prevalent, and its associated factors are mostly relational.

## Data Availability

The dataset supporting the findings of this study can be made available upon request to the first author whose email is *mksutete@gmail.com*

## Abbreviations

EPDS: Edinburgh Postnatal Depression Scale
IPV: Intimate Partner Violence
LMICs: Low- and middle-income countries
PID: Participant Identity Number
SCID-IV: Structured Clinical Interview for Diagnostic and Statistical Manual 4th edition
